# The gut-liver axis modulates intestinal immune homeostasis

**DOI:** 10.1016/j.mucimm.2025.12.001

**Published:** 2025-12-21

**Authors:** Yang Gu, Chuan Wu

**Affiliations:** Experimental Immunology Branch, National Cancer Institute, National Institutes of Health, Bethesda, MD 20892, USA

## Abstract

The intestine and liver are physically interconnected through the biliary system, portal circulation, lymphatic network, and neural pathways, collectively forming the gut–liver axis. The gut–liver axis and the immune system are engaged in a complex regulatory relationship. Over the past few decades, significant progress has been made in elucidating how liver-derived bile acids (BAs) shape intestinal immunity through interactions with the gut microbiota. However, the multidirectional regulatory pathways by which the liver orchestrates intestinal immune homeostasis remain incompletely defined. In this review, we highlight liver-derived cues—including the BAs, neural signals, nutrient metabolism, hormones, hepatically secreted proteins, and the complement system—and their impact on intestinal innate and adaptive immune cells. Furthermore, we discuss how intestinal dysbiosis contributes to the progression of liver inflammation and hepatocellular carcinoma (HCC) via immune cells. A comprehensive understanding of these intricate interactions may uncover novel therapeutic strategies for treating gut- and liver-associated immune disorders.

## Introduction

The gut-liver axis represents a complex and dynamic bidirectional communication network that plays a crucial role in maintaining systemic immune homeostasis. In the gut-to-liver direction, disruption of intestinal immune barrier allows an increased burden of microbe-associated pathogen-associated molecular patterns (PAMPs) to enter the portal circulation beyond physiological levels, thereby promoting liver inflammation and contributing to disease progression.^1,2^ For example, alcohol feeding triggers intestinal inflammation through increased TNF-α-producing monocytes and macrophages, which activate TNFR1 on intestinal epithelial cells, resulting in increased intestinal barrier permeability that facilitates microbial products such as lipopolysaccharide (LPS) translocation and subsequent induction of alcoholic liver disease.^3^

Conversely, the liver serves as an active hub modulating intestinal homeostasis. The liver regulates intestinal stem cell renewal^4^ and produces BAs—released into the duodenum via the biliary system—that modulate intestinal immune cell function.^5,6^ Additionally, the gut-liver axis regulates gut physiology via neural-immune interactions.^7^ Despite these insights, the extent to which the liver regulates intestinal immunity through an additional, yet critical, layer of control has not been systematically reviewed.

In this review, we summarize the roles of liver-derived factors—including, BAs, neural signals, nutrient metabolism, hormones, hepatokines, and the complement systems—in regulating intestinal innate and adaptive immune cells, highlighting their broad impact on intestinal immunity. Perturbations in these regulatory pathways have been implicated in the pathogenesis of inflammatory bowel disease (IBD), gut barrier dysfunction, and systemic immune imbalance. Elucidating these mechanisms may pave the way for novel therapeutic strategies targeting the gut-liver axis to restore immune homeostasis in both gastrointestinal and systemic diseases.

## Anatomical connections of the gut-liver axis

The gut-liver axis is maintained by a complex network of anatomical structures that enable continuous bidirectional communication between the two organs. This crosstalk is orchestrated through four major physiological pathways: the biliary system, the portal circulation, the lymphatic network, and neural pathway. The biliary tract transports BAs and liver-derived bioactive molecules into the intestinal lumen, influencing digestion, immunity, and microbial composition. The portal venous system delivers nutrients and microbial products from the gastrointestinal tract to the liver via the bloodstream, providing a direct route for metabolic and immune interactions. The mesenteric and hepatic lymphatic systems converge at the celiac lymph nodes (CLN), where processed gut-derived materials from the mesenteric lymph nodes (MLNs) integrate with hepatic lymphatic drainage, establishing a unified circulation pathway that facilitates immune crosstalk and systemic surveillance within the gut-liver axis. Finally, neural pathways provide rapid signaling in the gut-liver axis, integrating metabolic, immune, and behavioral responses through autonomic and sensory circuits. Together, these interconnected systems establish the structural foundation for the dynamic immunological interplay that defines the gut-liver axis.

### Biliary system

The biliary system constitutes a vital anatomical and functional bridge within the gut-liver axis. Bile is synthesized by hepatocytes and secreted into bile canaliculi, which converge into intrahepatic ducts, then drain into the common bile duct via the hepatic ducts.^8^ It is ultimately released into the duodenum through the ampulla of Vater, enabling direct communication between the liver and the intestinal lumen.^9^ Beyond their classical role in lipid emulsification and absorption of fat-soluble vitamins, BAs serve as important antimicrobial factors that shape the intestinal microecology. Through their amphipathic detergent properties, BAs directly disrupt bacterial cell membranes.^10^ In addition, some BAs serve as farnesoid X receptor (FXR) ligands to induce antimicrobial gene expression (e.g., IL-18) in ileal enterocytes,^11^ thereby maintaining the small intestine’s low bacterial burden that is essential for normal digestive function and prevention of bacterial translocation. As endocrine-like molecules, BAs also regulate intestinal immune homeostasis and systemic metabolism.

In addition to BAs, the biliary tract transports a variety of bioactive molecules, including phospholipids (e.g., lecithin),^12^ immunoglobulins (e.g., sIgA),^13^ and cholesterol,^14^ which may modulate gut microbiota composition and intestinal homeostasis. Experimental models of cholestasis, such as bile duct ligation, have shown that impaired bile flow disrupts gut barrier integrity, alters microbial communities, and promotes liver inflammation, underscoring the biliary system’s role in bidirectional immune regulation.^15^ Although the bile duct is anatomically a unidirectional channel, pathological conditions such as infection or intestinal dysbiosis may allow retrograde movement of microbial metabolites or pathogens into the biliary tree, triggering hepatic immune responses.^16^

### Portal circulation

The liver, as an immune metabolic organ,^17^ is uniquely positioned at the interface of systemic and intestinal circulation through its dual blood supply. Approximately 75 % of hepatic blood flow is delivered via the portal vein, which drains the gastrointestinal tract, pancreas, and spleen, while the remaining 25 % comes from the oxygen-rich hepatic artery.^18^ This vascular arrangement forms the anatomical and functional foundation of the gut–liver axis. Venous blood from the small intestine enters the superior mesenteric vein (SMV), whereas the colon and rectum are drained by the inferior mesenteric vein (IMV).^19^ These veins, along with the splenic and pancreatic veins, merge to form the portal vein, which delivers nutrient-rich and microbial metabolite-laden blood directly to the liver via the portal triads.^20^ Within the hepatic sinusoids—a specialized, low-pressure capillary network—portal and arterial blood mix and perfuse the lobular microenvironment, coming into intimate contact with Liver Sinusoidal Endothelial Cells (LSECs), Kupffer cells, and hepatic stellate cells. Dendritic cells (DCs), primarily localized in periportal regions,^21^ complement this sinusoidal surveillance by capturing gut-derived antigens and presenting them to lymphocytes.^22^ Processed blood then exits through the central veins, hepatic veins, and finally re-enters systemic circulation via the inferior vena cava.^23^ This complex vascular organization renders the liver not only a metabolic filter for intestinal inputs but also an essential immunological barrier that helps preserve host–microbiota homeostasis. Importantly, accumulating evidence indicates that liver-derived factors reach the intestine through the bloodstream and modulate immune cell differentiation and intestinal stem cell expansion.^4,24^ For instance, the liver-secreted factor Pigment Epithelium Derived Factor (PEDF) enters the systemic circulation via the hepatic vein and is subsequently delivered to the intestinal mucosa. PEDF has been shown to directly bind the Wnt co-receptors LRP5/6, blocking Wnt ligand-induced receptor complex formation^25^ and LRP6 phosphorylation,^26^ a critical step for Wnt signal transduction. By antagonizing LRP5/6 function, liver-derived PEDF inhibits Wnt/β-catenin signaling to restrain excessive intestinal stem cell self-renewal, thereby maintaining gut homeostasis.^4^

### Lymphatic networks

The lymphatic network functions as a ’messenger highway’ for the immune system, transporting immune signals from peripheral tissues to lymph nodes for immune surveillance and response initiation. These organs are where mature lymphocytes encounter antigens, triggering immune activation and differentiation.^27^

Anatomically, the intestinal lymphatic system includes the lacteals, submucosal lymphatic vessels, and MLNs, which drain through a series of collecting vessels and lymphatic trunks before reaching the thoracic duct.^28^ Lacteals, located at the center of small intestinal villi, absorb chylomicrons, microbial metabolites, and luminal antigens. These contents are then drained into larger submucosal lymphatics and subsequently transported to the MLNs.^28,29^ Efferent lymphatics from the MLNs drain into the superior mesenteric lymph nodes and subsequently converge to form the intestinal lymphatic trunk. This trunk, together with the right and left lumbar trunks, unites at the cisterna chyli, which gives rise to the thoracic duct. The thoracic duct ascends through the thorax and drains into the venous system at the left venous angle, formed by the junction of the left subclavian and internal jugular veins, allowing lymph to re-enter the systemic circulation.^30^

The liver is the largest lymph-producing organ, generating 25–50 % of thoracic duct lymph flow.^31^ Hepatic lymph originates from plasma ultrafiltration through fenestrated LSECs into the space of Disse.^32^ In mice, hepatic lymphatic drainage is mediated by two anatomically discrete and functionally specialized lymph nodes: the portal lymph node (PLN) and CLN, both situated in proximity to the hepatic hilum yet receiving lymph through independent afferent pathways.^33,34^ Anatomical studies have revealed shared lymph nodes between the liver and the duodenum, likely due to their common embryonic origin in the ventral foregut and parallel lymphatic development during organogenesis.^35^ Consistently, accumulating studies showed that the CLN drains multiple organs—not only the liver and upper duodenum but also the pancreas—whereas the PLN is limited to draining only the liver and pancreas.^33^ Although lymph flow is strictly unidirectional—precluding retrograde flux into parenchymal tissues—the convergent drainage pattern enables antigen-presenting cells from liver and intestine to co-localize within CLN microdomains, present their respective antigenic repertoires to shared T cell pools, and engage in bidirectional immune modulation through juxtacrine and paracrine signaling mechanisms.^33,35^ For example, studies in murine models have shown that duodenal viral infection may induce interferon-stimulated gene programs in liver- and pancreas-derived migratory DCs within gut-shared CLNs through paracrine signaling, even though these DCs themselves are uninfected.^35^ This lymphatic convergence thereby provides an anatomical substrate for gut-liver immune crosstalk in health and disease.

Liver-draining lymph nodes (the CLNs and PLNs), appear to contribute to adaptive immunity during gut-derived parasitic infections.^33^ Notably, the MLNs and CLNs were found to coordinate early T helper 2 cell (Th2) responses and the generation of IgG1 plasma cells, while PLNs may support T follicular helper cell (Tfh)-driven germinal center formation and antibody affinity maturation.^33^ Although MLN-derived CD4^+^ T cells have been shown to migrate to the liver and exacerbate hepatocellular damage in Metabolic dysfunction-Associated Steatotic Liver Disease (MASLD),^36^ the molecular or anatomical mechanisms underlying such inter-organ lymphatic migration remain poorly understood. This raises the possibility of therapeutically targeting shared lymphatic drainage routes to modulate gut–liver immune interactions in future research. In contrast to the well-mapped vascular routes, the lymphatic connections between the gut and liver remain poorly defined. Lymph flow is slow and does not directly reach hepatocytes, and tracing lymphatic pathways is technically challenging due to the lack of effective tracking tools and suitable animal models. These limitations have collectively rendered the “gut–liver lymphatic axis” a relatively underexplored area in immunology.

### Neural pathways

The peripheral nervous system comprises two major branches: the somatic nervous system and the autonomic nervous system.^37^ The somatic branch is under conscious control and primarily regulates skeletal muscle movement, while the autonomic branch operates independently of conscious control, modulating the activity of visceral organs such as the gastrointestinal tract and liver. The autonomic nervous system consists of the sympathetic and parasympathetic divisions,^38^ which regulate metabolic and immune homeostasis under ‘stress’ and ‘rest’ conditions, respectively.^39^ The vagus nerve (VN), a component of the parasympathetic nervous system, is currently the primary and most extensively studied neural pathway that directly connects the gut and liver.^40^

The VN comprises both efferent and afferent fibers, with afferent fibers constituting the majority. Efferent branches project to the enteric nervous plexuses^41^ and innervate the bile ducts and hepatic vasculature in the portal region via the hepatic plexus,^42^ regulating liver metabolism and immune responses. Afferent fibers transmit chemosensory information—such as nutrient status, microbial metabolites, and inflammatory signals—from the gut and hepatic portal area to the brainstem through the nodose ganglion, enabling the central nervous system to monitor and respond to gut–liver metabolic and immune states.^42^ Recent studies have shown that the gut microbiota can modulate the vagal nerve function. For instance, sulfate-reducing bacteria such as *Desulfovibrionales* (including *Bilophila* and *Desulfovibrio*) activate intestinal VN to promote acetylcholine (ACh) release. This, in turn, inhibits CD8^+^ T cell activity via the cholinergic receptor CHRM3, facilitating HCC progression.^43^ Conversely, hepatic vagotomy in mouse models, which disrupts sensory afferents from the liver to the brainstem, leads to reduced colonic regulatory T cell (Treg) populations and heightened susceptibility to colitis.^7^ Additionally, vagal afferents innervating the portal/liver regions can sense diverse gut-derived signals, including GLP-1, to ameliorate dysregulated obesity, and diabetes.^44^

Despite its innervation, the liver’s sensory nerves are primarily confined to the capsule and portal regions, with few sensory branches extending into the parenchyma. This anatomical arrangement limits direct neural regulation of intrahepatic metabolism and immunity and may constrain studies on how enteric neural signals influence liver parenchymal function. In contrast, the enteric nervous system (ENS) autonomously controls intestinal motility, secretion, blood flow, and microbial sensing. Through its interactions with both the VN and sympathetic nerves, the ENS also coordinates with the central nervous system to mediate integrated gut–brain–liver communication.^42^

**Fig. 1.**
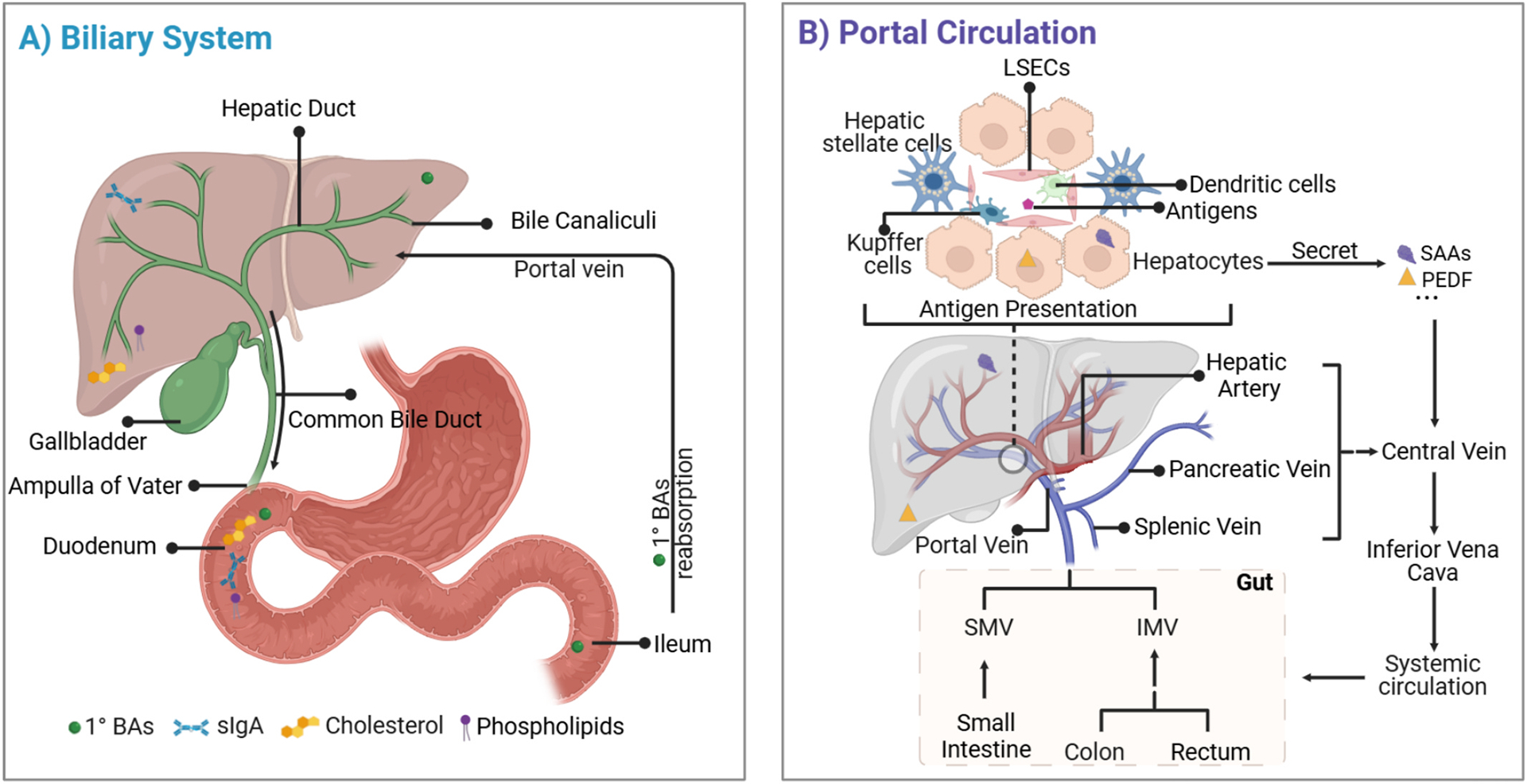
Anatomical connections of biliary system and portal circulation in the gut-liver axis. A). Biliary system: Bile acids (BAs), secretory IgA (sIgA), Phospholipids (lecithin), cholesterol, and other mediators are transported from the liver to the duodenum via bile duct, influencing intestinal homeostasis and microbiota composition. BAs are reabsorbed in the ileum and transported back to the liver through the portal circulation. B). Portal circulation: The portal circulation transports blood from the intestines (via SMV and IMV), spleen, and pancreas to the liver through the portal vein. Within hepatic sinusoids, LSECs, hepatic stellate cells, and Kupffer cells mediate antigen presentation and immune regulation, while dendritic cells perform similar functions in the portal areas. Hepatocytes secrete factors such as SAAs and PEDF regulate intestinal homeostasis. Processed blood exits via the central vein to systemic circulation through the inferior vena cava. SMV, superior mesenteric vein; IMV, inferior mesenteric vein; LSECs, liver sinusoidal endothelial cells; SAAs, serum amyloid A; PEDF, pigment epithelium-derived factor.

**Fig. 2.**
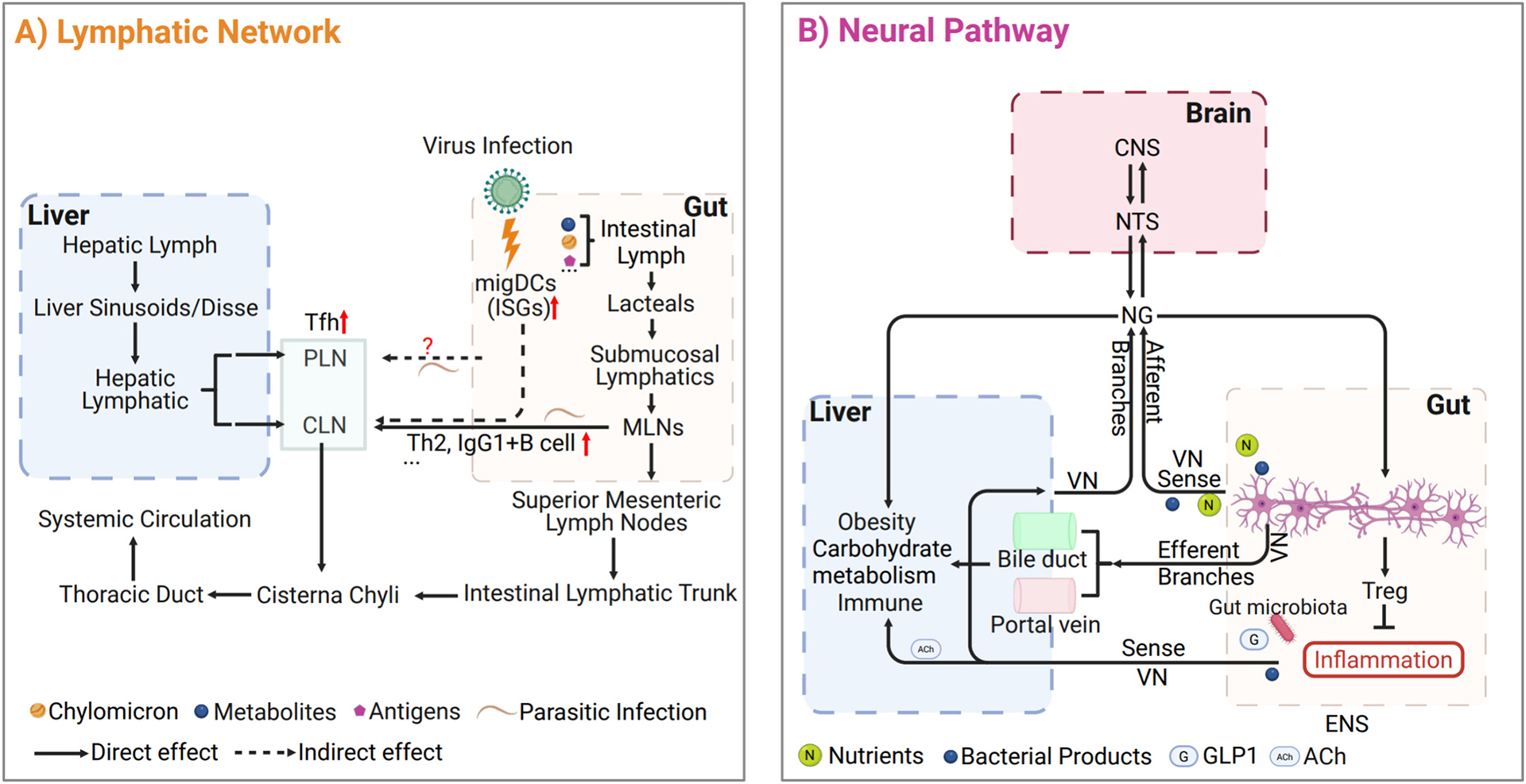
Anatomical connections of lymphatic network and neural pathway in the gutliver axis. A). Lymphatic network: Intestinal lymphatic fluid, including immune cells, antigens, metabolites, chylomicrons, and other components, flows from intestinal lacteals → submucosal lymphatics → mesenteric lymph nodes (MLNs) → superior mesenteric lymph nodes → intestinal lymphatic trunk → cisterna chyli → Thoracic duct → Systemic circulation. The hepatic lymphatic system drains from liver sinusoids/Disse spaces through hepatic lymphatic vessels to portal lymph nodes (PLN) and celiac lymph nodes (CLN), which converge at the cisterna chyli. Virus or parasitic Infection induces the expansion of various lymphocyte subsets such as migDC, Th2 cells and IgG1^+^ B cells in MLNs, which interact with hepatic lymphocytes in shared lymph nodes. This lymphatic communication enables gut-liver immune crosstalk. Tfh, T follicular helper cells; migDCs, migratory dendritic cells; ISGs, interferon-stimulated gene programs; MLNs, mesenteric lymph nodes; B). Neural pathway: The vagus nerve (VN) provides bidirectional communication between the gut, brain, and liver. Sensory VN fibers detect gut-derived signals including nutrients, bacterial products, GLP-1, immune signals and gut microbiota, transmitting them via the nodose ganglion (NG) to the nucleus tractus solitarius (NTS) and CNS. Efferent VN branches regulate hepatic and intestinal metabolism and immune function. This neural circuit enables rapid gut-brain-liver crosstalk. CNS, central nervous system; GLP-1, glucagon-like peptide-1; Treg, regulatory T cell; ACh, acetylcholine.

Together, these four anatomical pathways—the biliary system, portal circulation, lymphatic networks, and neural pathways—form a tightly integrated network that underlies the structural and physiological foundation of the gut–liver axis (Figs. 1, 2). Each connection enables not only material exchange but also immune communication, allowing the liver to continuously sense, respond to, and influence the intestinal environment.

## The gut–liver axis regulates intestinal immune cells

Intestinal immune homeostasis is maintained through coordinated innate and adaptive immunity. Innate immune cells, such as DCs, macrophages, and innate lymphoid cells (ILCs), provide rapid responses to environmental challenges, with DCs uniquely bridging innate recognition and adaptive immunity through professional antigen presentation. Adaptive immune cells, such as Tregs, T helper 17 (Th17) cells, and B cells, orchestrate antigen-specific responses that balance tolerance to commensals and immunity against pathogens. Accumulating evidence demonstrates that the liver profoundly influences both innate and adaptive intestinal immunity through multiple mechanisms. Liver-derived factors—such as BAs, serum amyloid A proteins (SAAs), complement components, and vitamins—reach the intestine via the biliary system and portal circulation, where they modulate immune cell differentiation, activation, and function. Additionally, the liver shapes intestinal immunity through neural signaling via the hepatic VN. In this section, we discuss how the liver shapes intestinal immune responses by regulating distinct innate and adaptive immune cell populations, highlighting the liver’s role in the gut–liver axis-mediated control of intestinal immunity.

### Regulation of innate immune cells

#### DCs

Intestinal DCs respond to various signals, including microbial components, food antigens, damage-associated molecular patterns (DAMPs) and cytokines from the local microenvironment.^45^ Activated DCs orchestrate T cell responses that determine the balance between inflammation and immune tolerance. Intestinal DCs are primarily located in the lamina propria, Peyer’s patches, and MLNs.^46^

**Fig. 3.**
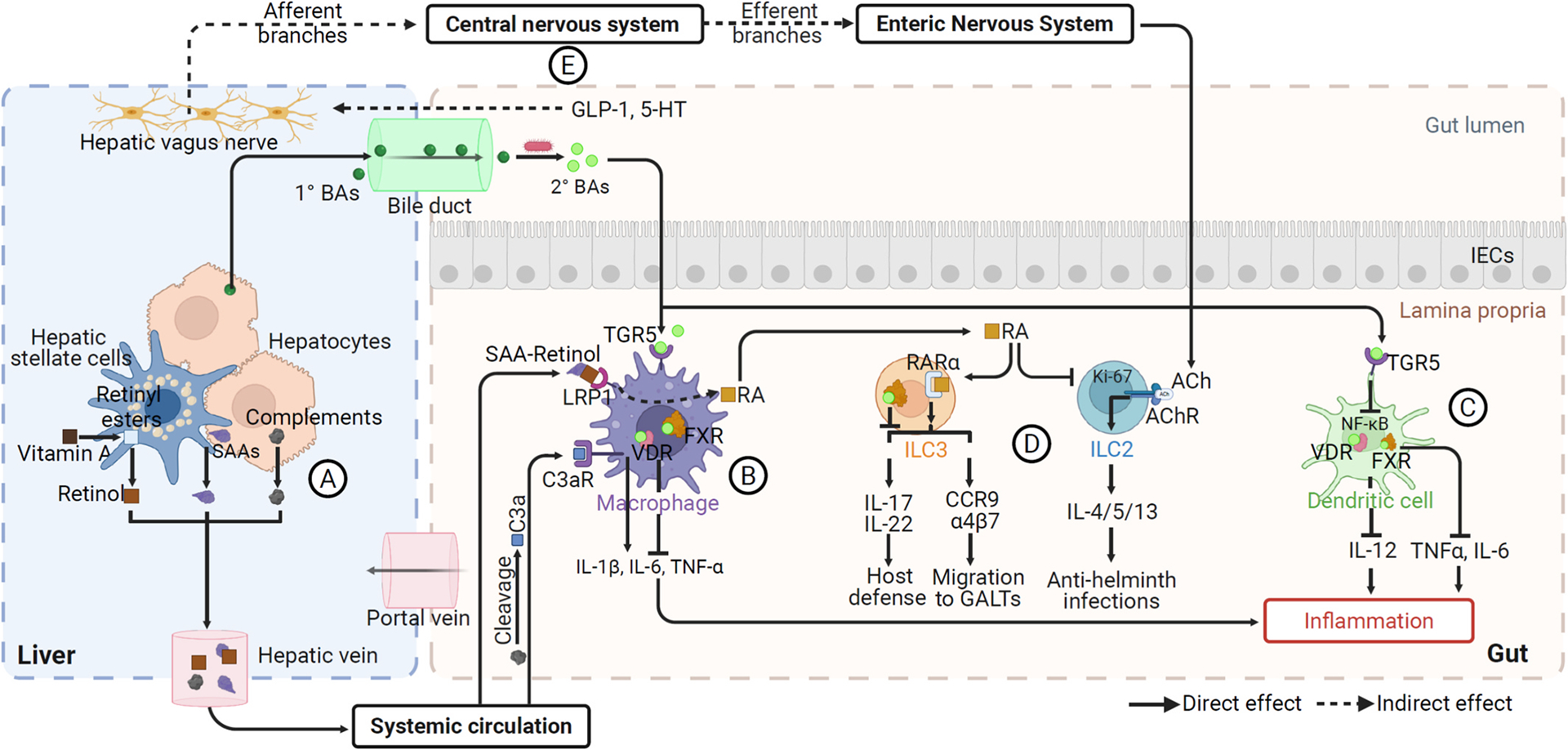
The gut-liver axis regulates intestinal innate immune cells A). The liver controls multiple pathways through SAA production, complement synthesis, and vitamin A storage and release. The liver modulates intestinal immune responses by controlling the systemic concentrations of these bioactive molecules. B). SAA serves a dual function as both an inflammatory mediator and a retinol transport carrier. The SAAretinol complex is recognized by LRP1 in intestinal macrophages, facilitating retinoic acid (RA) synthesis. Complement activation ultimately leads to the formation of C3a. Activated complement C3a promotes macrophage polarization toward the pro-inflammatory M1 phenotype and enhances TNF-α production, contributing to inflammatory responses. 2° BAs engage bile acid receptors—including TGR5, VDR, and FXR—to exert antiinflammatory effects, primarily by inhibiting macrophage secretion of pro-inflammatory cytokines (IL-1β, IL-6, and TNF-α). C). These 2° BAs promote the anti-inflammatory properties of DCs by suppressing IL-12 expression via TGR5 and VDR. Iso-DCA antagonizes FXR to suppress TNFα and IL-6 production. D). RA promotes ILC3 differentiation and IL-22 production while upregulating expression of intestinal homing markers CCR9 and α4β7 through retinoic acid receptor alpha (RARα). Conversely, RA suppresses ILC2 proliferation by downregulating Ki-67 expression. E). Hepatic vagal afferents serve as chemosensors for gut-derived signals, including GLP-1 and serotonin (5-HT), transmitting this information via the left nodose ganglion to the central nervous system (CNS). Subsequently, these processed signals are relayed through parasympathetic efferent nerves to modulate enteric nervous system (ENS) activity, establishing a hepato-enteric neural circuit. ENS-derived acetylcholine (ACh) engages AChR-expressing ILC2s to augment type 2 cytokine responses (IL-4/IL-5/IL-13), facilitating helminth clearance and intestinal immune homeostasis. GALTs: gutassociated lymphoid tissues.

DCs have been shown to highly express bile acid receptors, including vitamin D receptor (VDR) and Takeda G protein-coupled receptor 5 (TGR5).^47,48^ Activation of distinct bile acid receptors in intestinal DCs mainly orchestrates anti-inflammatory functions (Fig. 3). For instance, VDR, a ligand-activated nuclear transcription factor^49^ expressed in CD11c^+^ DCs, can be activated by its canonical ligand 1,25-dihydroxyvitamin D_3_ to inhibit DC maturation through downregulation of costimulatory molecules and IL-12 production, thereby promoting transplantation tolerance.^47^ TGR5, a cell surface receptor activated by BAs, plays important roles in energy metabolism and anti-inflammatory responses.^50^ Deoxycholic acid (DCA), a TGR5 agonist, inhibits CD11c^+^MHC class II (MHC-II)^high^ DC activation and reduces the severity of autoimmune inflammation through a defined signaling cascade. Mechanistically, TGR5 activation triggers adenylate cyclase, leading to increased intracellular cyclic AMP (cAMP) levels, which in turn activates protein kinase A (PKA).^51^ PKA activation subsequently suppresses NF-κB-mediated pro-inflammatory cytokine production in DCs, thereby attenuating inflammatory responses.^52^

DC responsiveness to BA signaling exhibits pathway-specific heterogeneity. The FXR is a ligand-activated nuclear receptor that, upon activation by BAs, translocates to the nucleus and functions as a transcription factor to regulate downstream gene expression.^53^ Isodeoxycholic acid (*iso*-DCA), a microbiota-derived BA, has been identified as a natural FXR antagonist in intestinal DCs. Inhibition of FXR by *iso*-DCA reduces the immunostimulatory capacity of DCs and downregulates inflammatory cytokine production such as TNFα and IL-6. This shift promotes a tolerogenic DC phenotype that favors Treg differentiation over Th17 in the colon.^54^ CD11c is a widely used surface marker expressed across multiple DC subsets, including conventional DC1 (cDC1, CD11c^+^MHC-II^+^CD103^+^CD11b^−^ XCR1^+^), cDC2s (CD11c^+^MHC-II^+^CD103^+^CD11b^+^SIRPα^+^), plasmacytoid DCs (pDCs, CD11c^int^MHC-II^low^B220^+^Siglec-H^+^), and monocyte-derived DCs (moDCs, CD11c^+^MHC^_^ II^+^CD11b^+^CD64^+^Ly6C^+^).^55,56^ Future investigations should systematically characterize BA receptor expression profiles across purified DC subsets and elucidate the molecular mechanisms by which different BAs modulate subset-specific DC functions, providing a more refined understanding of BA-DC crosstalk in intestinal immune homeostasis.

#### Macrophages

Macrophages are key components of the innate immune system, responsible for regulating inflammatory responses, clearing pathogens and apoptotic cells, and promoting tissue repair and regeneration.^57^ They exhibit plasticity and functional diversity, adopting context-dependent activation states along a spectrum from pro-inflammatory to anti-inflammatory and tissue-reparative phenotypes.^58^ Pro-inflammatory macrophages drive antimicrobial responses and inflammation, while anti-inflammatory and tissue-reparative macrophages support wound healing, fibrosis resolution, and immune homeostasis.^59^

Intestinal macrophages are directly regulated by hepatic proteins, including SAAs and complements (Fig. 3). SAAs are rapidly induced in the liver in response to inflammatory cues such as IL-1β, IL-6, and LPS,^60^ and are secreted into the circulation as a major acute-phase reactant that amplifies inflammation. Beyond their role in acute-phase responses, liver-derived SAAs also function as retinol-binding proteins, delivering retinol to intestinal myeloid cells via the endocytic receptor LRP1.^61^ Specifically, SAA-retinol complexes are captured by LRP1-expressing CD11c^+^MHCII^+^ myeloid cells, which include both F4/80^−^ DCs and F4/80^+^ macrophages in the intestinal lamina propria.^61^ These cells convert retinol to retinoic acid (RA) via retinaldehyde dehydrogenase (RALDH) enzymes.^62^ The RA-producing myeloid cells then migrate to MLNs, where they present antigens to naive T and B cells. During this process, RA diffuses into lymphocytes and binds to retinoic acid receptors (RARs) in the nucleus. Upon RA binding, RARs heterodimerize with retinoid X receptors (RXRs) and bind to retinoic acid response elements (RAREs) in target gene promoters,^63^ activating RA-dependent gene expression programs that induce the gut-homing receptors CCR9 and integrin α4β7.^64^ CCR9 interacts with its ligand CCL25, which is constitutively expressed by the intestinal epithelium, creating a chemotactic gradient that guides CCR9^+^ lymphocyte trafficking toward the small intestinal mucosa.^65^ Concurrently, integrin α4β7 binds to mucosal addressin cell adhesion molecule-1 (MAdCAM-1), which is selectively expressed on endothelial cells of lamina propria venules throughout the small and large intestine.^66^ These mechanisms promote the homing of CCR9^+^α4β7^+^ B and T cells from MLNs to the intestinal mucosa and, in the case of B cells, enhance IgA production.^61^ In parallel with retinol delivery via SAAs, the liver also modulates intestinal macrophages through the complement system. The majority (80–90 %) of plasma complement components are synthesized by hepatocytes.^67^ Most complement proteins are produced as inactive precursors and acquire biological activity only after proteolytic cleavage, generating functional fragments such as C3a, C3b, C5a, and C5b.^67^ Intestinal lamina propria F4/80^+^ macrophages express high levels of the C3a receptor (C3aR).^68^ Activation of C3aR by C3a leads to rapid receptor internalization and calcium influx, promoting macrophage polarization toward the proinflammatory phenotype and enhancing TNF-α production.^68,69^

BAs are also well known for directly regulating intestinal macrophages via FXR, TGR5 and VDR receptors. CDCA, a primary BA and ligand for FXR, suppresses the release of pro-inflammatory cytokines IL-1β, IL-6, and TNF-α from macrophages.^70^ TGR5 expression is elevated in lamina propria CD14^+^ macrophages from the inflamed intestinal mucosa of Crohn’s Disease (CD) patients compared to non-CD patients.^71^ Bacterial BA derivatives, such as deoxycholic acid (DCA) and lithocholic acid (LCA), act as TGR5 agonists that activate TGR5–cAMP signaling to suppress TNF-α production in inflammatory macrophages, suggesting that TGR5 signaling may serve as a potential therapeutic target for IBD.^71^ Other TGR5 agonists, such as 3-oxoLCA and isoLCA, have also been shown to exert anti-inflammatory effects on macrophages.^72^ These molecules suppress the expression of IL-6 and CD86—markers of proinflammatory macrophages^72^–thereby promoting macrophage polarization toward an anti-inflammatory phenotype. Additionally, 1,25-dihydroxyvitamin D has been shown to shift glucose-induced macrophage polarization from a pro-inflammatory phenotype to an anti-inflammatory phenotype.^73^ Although VDR can be activated by BAs like LCA, it is still unclear whether this activation recapitulates the anti-inflammatory effects of 1,25-dihydroxyvitamin D in intestinal macrophages.

#### Ilcs

ILCs play critical roles in infection control, immune regulation, and tissue repair.^74^ Due to their transcriptional and functional similarities with T helper cell subsets, ILC1s, ILC2s, and ILC3s are generally regarded as the innate counterparts of Th1, Th2, and Th17 cells, respectively. ILC1s react to intracellular pathogens, such as viruses, and to tumors, primarily through the secretion of IFN-γ; ILC2s respond to large extracellular parasites and allergens, producing IL-4, IL-5, and IL-13;^75^ ILC3s combat extracellular microbes, including fungi and bacteria, via production of IL-17 and IL-22.^76,77^ As tissue-resident cells, both ILC2s and ILC3s are present in the human intestine.^78^ ILC3s are the predominant population throughout most regions of the intestinal lamina propria, whereas ILC2s are specifically enriched in the lamina propria of the jejunum.^78^ In contrast, ILC1s are relatively rare in the intestinal mucosa under steady state, although their numbers can increase in response to inflammation.^79^ Given these distribution patterns, particular attention has been directed toward how the liver modulates intestinal ILC2s and ILC3s subsets (Fig. 3).

The intestine, as the primary site of nutrient absorption, and the liver, as the central organ for nutrient metabolism and storage, work in concert to maintain host nutrient homeostasis. Dietary micronutrients, such as various vitamins, can significantly modulate immune responses through ILC2s and ILC3s.^80^ Following dietary intake, vitamin A is converted into retinyl esters and primarily stored in hepatic stellate cells.^81^ Upon demand, retinyl esters are hydrolyzed into retinols, which are released into the circulation and taken up by intestinal immune cells such as DCs and macrophages.^61,82^ These cells enzymatically convert retinol into RA, the active metabolite of vitamin A.^61^ RA plays distinct regulatory roles in intestinal immunity: it suppresses IL-13–producing ILC2s by downregulating IL-7Rα, a key molecule required for ILC2 development and function, while promoting ILC3 development and IL-22 production, thereby enhancing mucosal defense against bacterial infections.^83^ Additionally, RA induces high expression of the gut-homing markers CCR9 and integrin α4β7 in ILC3s, facilitating their migration to the intestine.^84^

The liver also regulates intestinal ILCs by modulating systemic hormone clearance, the release of enteric neurotransmitters, and BA metabolites. Treatment of ILC2s with dihydrotestosterone (DHT), a potent androgen, significantly suppressed their pathogenic activation by reducing the release of proinflammatory cytokines, such as IL-13 and GM-CSF.^85^ Given the liver’s role in clearing circulating androgens,^86,87^ together with the high expression of androgen receptor in intestinal ILC2s,^85^ it is possible that the liver modulates intestinal ILC2s through androgen metabolism. Neuroimmune interactions have also emerged as key regulators of ILC2s. Selective hepatic vagotomy reduces local ACh levels in the colon via effects on enteric neurons.^7^ Since ILC2s express AChRs, they can be activated by ACh to enhance cytokine production, promoting helminth expulsion.^88^ Thus, the gut-liver axis may control intestinal ILC2 function via vagal nerve signaling and ACh. ILC function is also subject to regulation by BA. Activation of FXR attenuates CCR6^+^ and CD4^+^ ILC3-associated intestinal inflammation by suppressing *Il17* and *Il22* expression.^89^

### Regulation of adaptive immune cells

#### Tregs

Tregs are essential for maintaining intestinal immune tolerance. The role of liver-derived BAs in regulating intestinal Tregs has been extensively investigated.^90^ Primary BAs synthesized in the liver are secreted into the duodenum through the biliary system. Approximately 95 % are reabsorbed in the ileum, while the remaining 5 % enter the colon for microbial transformation into secondary BAs.^91^ Although the direct regulatory effects of primary BAs on Tregs have not been fully explored, their metabolites-secondary BAs-have been shown to modulate Treg function.

**Fig. 4.**
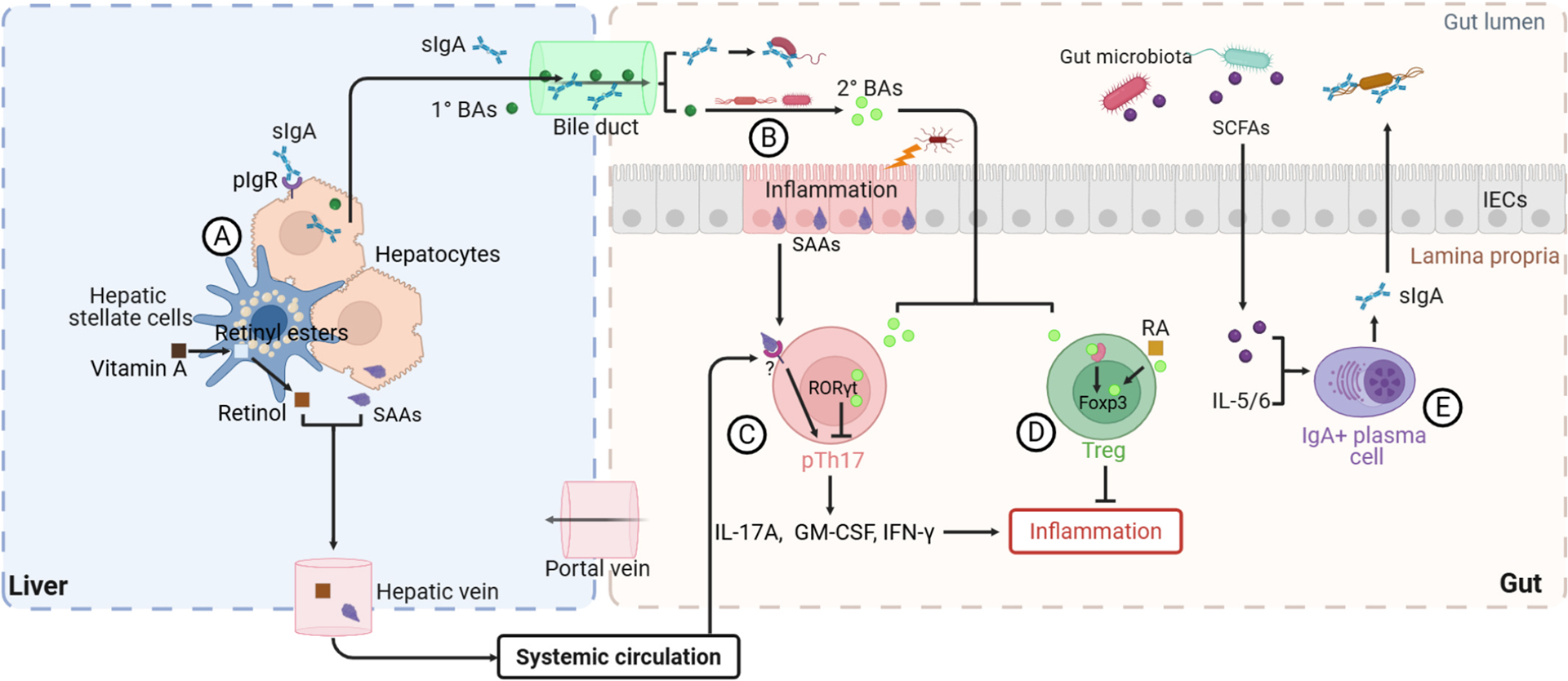
The gut-liver axis regulates intestinal adaptive immune cells. A). Hepatic stellate cells store retinyl esters and enzymatically release retinol into systemic circulation via hepatic veins. Meanwhile, hepatocyte-synthesized serum amyloid A proteins (SAAs) reach intestinal tissues through the bloodstream, while plasma IgA undergoes transcytosis through hepatocytes via the polymeric immunoglobulin receptor (pIgR) and is secreted into bile for delivery to the duodenum. B). Bile-transported IgA and locally secreted IgA from intestinal plasma cells coat commensal microbiota to maintain immune homeostasis and shape the microbial landscape. Simultaneously, gut microbiota metabolize primary BAs (1° BAs) into secondary BAs (2° BAs), which enter the lamina propria and modulate immune cell functions. C). Liver-derived SAA can promote the differentiation of pathogenic Th17 (pTh17) cells by upregulating T-bet, GzmB, and IFN-γ, exacerbating intestinal inflammation. Additionally, upon segmented filamentous bacteria (SFB) infection, intestinal epithelial cells have also been found to produce SAAs, which induce pTh17 differentiation. Furthermore, 2° BAs, such as 3-oxoLCA and isoLCA, directly bind to RORγt and attenuate its transcriptional activity, leading to reduced IL-17A production. D). Intestinal myeloid cells metabolize retinol to retinoic acid (RA), which enhances Treg differentiation and immune tolerance. Concurrently, 2° BAs promote Treg development via Foxp3 upregulation, establishing complementary pathways for maintaining intestinal immune homeostasis. E). The gut microbiota produces short-chain fatty acids (SCFAs), which promote plasma cell differentiation and enhance the production of IgA and IgG. Intestinal DC-mediated vitamin A metabolism generates RA, which induces IL-6/IL-5 production to drive IgA^+^ plasma cell differentiation.

In murine models, microbial BA metabolite isoallo-lithocholic acid (isoallo-LCA) enhances Treg differentiation in the colon. Isoallo-LCA increases mitochondrial reactive oxygen species (mitoROS) production in Treg, which promotes H3K27 acetylation at the Foxp3 promoter, facilitating chromatin opening and Foxp3 transcription, thereby driving Treg differentiation.^92^ In addition, isoallo-LCA directly binds to the Foxp3 enhancer, known as conserved noncoding sequence 3 (CNS3), promoting Foxp3 transcription and Treg differentiation (Fig. 4).

Beyond direct epigenetic modulation by BAs, Treg regulation is also influenced by bile acid receptors. FXR plays a critical role in BA metabolism, lipid metabolism, and inflammatory responses.^93^ Iso-DCA, a microbiota-derived BA, acts as a natural FXR antagonist to promote retinoic acid receptor-related orphan receptor gamma t (RORγt)^+^ Treg generation in the colonic lamina propria through modulating DC function.^54^ Consistently, FXR-deficient DCs exhibit enhanced baseline Treg induction capacity that is not further augmented by *iso*-DCA treatment.^54^ These findings provide evidence that liver metabolites regulate Tregs through interactions with the gut microbiota.

The VDR, upon activation by its canonical ligand 1,25-dihydroxyvitamin D_3_—an endogenous hormone—translocates into the nucleus, where it interacts with vitamin D response elements (VDREs) in the Foxp3 promoter region.^94^ This interaction enhances Foxp3 transcription and Tregs proportion.^94^ In addition to its classical ligand, VDR can also be activated by LCA, a secondary BA identified as a non-classical agonist.^95^ However, it remains unclear whether LCA can regulate intestinal Treg differentiation via VDR. Moreover, BA secretion into the intestine is triggered by feeding,^96^ and feeding behavior is under circadian control.^97^ Given that BAs regulate intestinal Tregs and that Tregs in visceral adipose tissue (VAT) display circadian rhythmicity,^98^ it is plausible that intestinal Tregs are subject to circadian regulation mediated by feeding-entrained fluctuations in BAs.

Colonic Tregs are also regulated by neural pathways along the liver-brain-gut axis. Hepatic vagal afferent nerves sense the enterohepatic microenvironment and transmit neural signals to the nucleus tractus solitarius (NTS) in the brainstem, which in turn modulates vagal parasympathetic output and enteric neuronal activity.^99^ In hepatic vagotomy mouse models, which disrupt left vagal sensory afferents from the liver to the brainstem, colonic Tregs are significantly reduced, leading to increased susceptibility to colitis.^7^ Mechanistically, hepatic vagal sensory afferents promote the secretion of ACh from enteric neurons, which stimulate muscarinic acetylcholine receptors (mAChR) on CX3CR1^+^ mononuclear phagocytes to generate RA locally within the lamina propria, thereby supporting the generation of the colonic RORγt^+^ Treg pool that respond to microbiota.^7,100^ Complementing this local induction pathway, CCR7 mediates intestinal DC migration to gut lymph nodes,^101^ where they express RALDH enzymes that convert retinol to RA.^102^ RA synergizes with TGF-β to promote differentiation of naive CD4^+^ T cells into Foxp3^+^ Tregs while simultaneously inhibiting the proinflammatory Th17 program.^103^ Notably, this lymph node-based Treg induction exhibits anatomical compartmentalization, with a gradient declining from proximal to distal regions along the intestinal tract. Under homeostatic conditions, duodenal-draining lymph nodes serve as the primary sites for Foxp3^+^ Treg induction in response to dietary antigens.^104^ Beyond its direct effects on Treg induction, RA imprints gut-homing properties on T cells by inducing expression of α4β7 integrin and CCR9, enabling their migration to intestinal tissues.^64,102^ Moreover, RA directly induces CD161 expression on Tregs through activation of RARs in humans. CD161 ligation enhances TCR signaling and production of wound-healing cytokines including IL-10 and IL-22 that accelerate intestinal epithelial repair and reduce inflammation in CD.^105^

#### Th17 cells

Th17 cells are a subset of CD4 + T cells characterized by the expression of the transcription factor RORγt,^106^ and the secretion of IL-17 and IL-22.^107^ Th17 cells can be further categorized into two subsets based on their inflammatory potential: pathogenic and non-pathogenic Th17 cells.^108^ Besides IL-17 production, pathogenic Th17 cells produce potent pro-inflammatory cytokines such as IFN-γ^109^ and GM-CSF,^108^ contributing to the development of autoimmune diseases. In contrast, non-pathogenic Th17 cells secrete immunoregulatory cytokines like IL-10 and exhibit anti-inflammatory properties.^110^

Emerging evidence suggests that intestinal Th17 cells are directly regulated by the liver through liver-derived factors and BAs. SAAs play a context-dependent role in Th17 differentiation. Under homeostatic conditions, commensal bacteria such as segmented filamentous bacteria (SFB) induce SAA production, which promotes Th17 cell differentiation without eliciting pathology, contributing to mucosal immunity.^111,112^ However, during sepsis and/or systemic inflammation, SAAs are prominently induced in the liver, with serum concentrations rising up to 1,000-fold above baseline levels.^113,114^ Under these inflammatory conditions, recombinant SAAs have been shown to induce a pathogenic Th17 transcriptional program, characterized by the upregulation of genes associated with chronic inflammatory diseases.^61,115^ Consistently, liver-specific overexpression of SAA increases pathogenic Th17 proportion in experimental autoimmune encephalomyelitis (EAE) model.^24^ Given that pathogenic Th17 cells aggravate intestinal inflammatory diseases via upregulating T-bet, GzmB and IFN-γ,^24^ these findings may partially explain the clinical observation of elevated serum SAA levels in patients with CD or ulcerative colitis (UC)^116^ (Fig. 4). Additionally, in SFB-colonized mice, intestinal epithelial cells were also found to produce SAAs, indicating a potential local source of SAAs in the gut.^111,115^ Further studies are needed to distinguish between systemic and local regulation of intestinal Th17 responses. The specific receptor mediating SAA effects on Th17 cells remains unclear. Although several receptors have been proposed, including TLR2 and FPRL-1,^113^ genetic knockout of these receptors did not impair SAA-induced pathogenic Th17 differentiation.^24^

Intestinal Th17 cells are also directly regulated by BAs. 3-oxoLCA and isoLCA, secondary BAs metabolites derived from gut microbial oxidation of LCA–suppress Th17 cell differentiation by directly binding to RORγt and attenuating its transcriptional activity, reducing IL-17A production.^92,117^ Ursodeoxycholic acid (UDCA) restricts Th17 cell function by downregulating IL-17A and TNF-α expression in goat models of high-fat diet–induced Metabolic dysfunction-Associated Steatohepatitis (MASH).^118^ Exposure of Th17 cells to NorUDCA, modified based on UDCA, dampens their pathogenicity and expansion in the intestine,^119^ highlighting its therapeutic potential for treating Th17-mediated intestinal inflammation.

#### B cells

Intestinal B cells—particularly plasma cells residing in the lamina propria, Peyer’s patches, and MLNs—play a pivotal role in maintaining mucosal immunity by producing secretory IgA (sIgA).^120^ Emerging evidence suggests that the liver may influence the differentiation of intestinal B cells and the production of IgA through multiple mechanisms (Fig. 4).

Serum-derived polymeric IgA (pIgA) can be internalized by hepatocytes or cholangiocytes via the polymeric immunoglobulin receptor (pIgR) and subsequently secreted into the small intestine through bile, especially under conditions of microbial stress or inflammation.^13,121^ This liver-derived IgA may contribute to intestinal humoral homeostasis independently of mucosal B cells. In addition, the liver regulates intestinal B cell function through its role in nutrient metabolism. Vitamin A, primarily stored as retinyl esters in hepatic stellate cells in humans and mice,^81^ is essential for intestinal B cell development. Vitamin A deficiency leads to a marked reduction in intestinal B cells, as observed in both human and murine studies.^122^ Mechanistically, vitamin A is absorbed and metabolized by intestinal DCs into RA, which, in turn, induces the production of IL-6 and IL-5 to promote the differentiation of IgA^+^ plasma cells.^122^

Furthermore, the liver exerts an indirect influence on intestinal B cells by modulating the gut microbiota and its metabolites. Microbiota-derived short-chain fatty acids (SCFAs), such as acetate and butyrate, have been shown to promote B cell differentiation and enhance the production of IgA and IgG through epigenetic regulation, a mechanism conserved between humans and mice.^123^ Given that liver-derived BAs profoundly affect the composition of the gut microbiota in humans and mice,^124,125^ it is plausible that BAs influence intestinal B cell function by modulating SCFA production. Although intestinal B cells express limited bile acid receptors, it remains unclear whether BAs can directly regulate gut humoral responses.

Together, these findings highlight the liver’s multifaceted roles in shaping intestinal immunity. Through its metabolic, neural, and endocrine functions, the liver profoundly influences both innate and adaptive immune cell populations in the intestine. As a central immunometabolic hub of the gut–liver axis, the liver not only processes microbial and dietary signals but also orchestrates downstream immune responses, maintaining mucosal homeostasis and immune tolerance.

## The gut–liver axis in the liver diseases

Liver diseases encompass a spectrum of conditions, including MASLD, MASH, alcohol-associated liver disease, fibrosis, cirrhosis, and HCC, which may occur sequentially or coexist depending on etiology.^126,127^ Recent studies have highlighted the gut–liver axis as a critical regulator of liver diseases. For instance, germ-free mice show significant alterations in hepatic gene expression, revealing that microbiota influence hepatic transcriptional regulation.^128^ The translocation of microbes or their antigens, microbial metabolites such as SCFAs, lactate, and BAs, as well as the migration of intestinal immune cells, collectively contribute to the progression of liver diseases.

While low-level microbial translocation occurs physiologically at steady state as part of normal immune surveillance,^129^ the liver has evolved specialized defense mechanisms to intercept translocated enteric pathogens. Hepatic Kupffer cells exhibit preferential localization within the periportal region—the first site of contact for gut-derived blood via the portal vein—to provide immunological defense against enteric pathogens such as *Listeria monocytogenes*.^130^ This strategic positioning enables rapid clearance of translocated microbes before they can disseminate systemically. However, pathological conditions significantly enhance microbial translocation, overwhelming these defense mechanisms. Disruption of the intestinal epithelial and vascular barriers in disease states facilitates excessive microbial translocation, allowing increased amounts of bacteria and their components to reach the liver. These elevated levels of translocated microbes activate Kupffer cells and other hepatic immune populations via TLRs, exacerbating liver inflammation and fibrosis.^131,132^ On the other hand, mucosa-adapted strains such as *Enterococcus gallinarum* and *Lactobacillus reuteri* can evade immune clearance, gain enhanced translocation capacity under pathological conditions, and persist within the liver, resulting in chronic hepatic inflammation.^133^

Microbial metabolites—particularly SCFAs like butyrate, lactate and secondary BAs—serve as key mediators of gut–liver communication. Butyrate reaches the liver via the portal vein at physiologically relevant concentrations (10–50 μM),^134^ and exerts anti-inflammatory effects through several mechanisms. First, butyrate enhances prostaglandin E2 (PGE2) production while suppressing TNF secretion in Kupffer cells.^135^ Second, in MASLD, butyrate upregulates PPARα expression through histone H3K9 acetylation at its promoter. Elevated PPARα then promotes fatty acid β-oxidation to reduce lipid accumulation and suppresses NF-κB-mediated inflammation via transrepression, directly binding to the p-p65 and inhibiting its transcriptional activity. These dual metabolic and anti-inflammatory actions reduce hepatic steatosis and inflammation.^136^ Beyond SCFAs, gut microbiota-derived D-lactate reaches the liver at high concentrations via the portal vein and directly enhances Kupffer cell function by promoting both circulating pathogen capture and ROS-dependent bactericidal activity, thereby maintaining the liver’s immune surveillance against bloodstream infections.^137^ Antibiotic-induced dysbiosis or germ-free conditions abolish this protective effect, highlighting the indispensable contribution of gut microbiota-derived D-lactate to hepatic antimicrobial defense.^137^ Secondary BAs also regulate hepatic inflammation. For example, UDCA therapy reduces the expression of MHC I/II molecules on bile duct epithelial cells in patients with primary biliary cholangitis, preventing T-cell-mediated hepatocellular necrosis.^138^

Beyond metabolites, intestinal immune cells such as invariant natural killer T (iNKT) cells can migrate to the liver and promote hepatocyte apoptosis, exacerbating liver damage in alcohol-associated liver disease.^139^ IL-10 prevents the migration of intestinal CD4^+^α4β7^+^ T cells to the liver, inhibiting hepatitis.^140^ It is well established that T cells acquire gut-homing properties under the influence of RA produced by intestinal DCs.^141^ Interestingly, LSECs also induce gut-homing markers upregulation (α4β7 and CCR9) on naïve CD4^+^ T cells via RA signaling.^141^

Intestinal dysbiosis impairs anti-tumor immune surveillance and promotes the progression of liver diseases toward HCC.^142^ Loss of *Akkermansia muciniphila* leads to TLR4-dependent expansion of immunosuppressive myeloid-derived suppressor cells (MDSCs) and diminished T cell–mediated anti-tumor immunity in the liver.^142^ Mechanistically, *A. muciniphila* enhances intestinal barrier integrity by regulating mucus production and increasing SCFA levels, limiting the translocation of microbial products to the liver.^142^ Severe gut dysbiosis in patients with cirrhosis or HCC may contribute to poor responses to immunotherapy, making microbiota modulation a particularly promising strategy to improve treatment outcomes in liver cancer compared to other malignancies.^126^ In summary, the gut–liver axis integrates microbial, metabolic, and immune signals to maintain hepatic homeostasis, and its disruption contributes to the pathogenesis of liver diseases, from steatosis to HCC.

## Future directions

The gut-liver axis represents a frontier in mucosal immunology, with hepatic regulation of intestinal immunity emerging as a particularly promising yet underexplored area. While the microbiome’s impact on liver function has been extensively characterized,^143,144^ less is known about the reciprocal regulation, how the liver shapes intestinal immune responses. This review highlights several exciting breakthroughs in our understanding of how the liver modulates intestinal immune balance through multiple pathways, including BAs, neural signals, nutrient metabolism, hormones, hepatically secreted proteins, and the complement system.

Although emerging evidence underscores the roles of BAs and hepatic proteins in modulating gut immunity, key mechanistic details—such as the specific target cell subtypes, transport pathways, recognition mechanisms, and spatiotemporal expression dynamics—remain poorly defined. Recent technological advances offer unprecedented opportunities to dissect gut-liver-immune circuits at molecular resolution. Single-cell and spatial transcriptomics can pinpoint which intestinal cell subtypes express receptors for hepatic signals and where they localize anatomically, while integrating metabolomic tracing and temporal profiling can reveal how these signals traverse transport barriers and dynamically regulate immune responses over time. These high-resolution approaches should be deployed within physiologically integrated experimental systems. Animal models remain indispensable for studying the full complexity of inter-organ communication involving blood flow, barrier function, neural innervation, and systemic feedback loops. Rather than replacing animal models, emerging human-relevant platforms such as multi-organoid co-cultures or gut-liver-on-chip systems should serve as complementary tools—ideal for mechanistic validation, drug screening, and testing human-specific hypotheses generated from in vivo discoveries.

One of the most exciting frontiers is the neural dimension of gut-liver-immune communication. Unlike humoral signals that diffuse slowly through tissues and circulation, neural circuits enable rapid coordination.^145^ Yet we know remarkably little about how hepatic vagal afferents sense immune status and relay information to brainstem nuclei that, in turn, modulate gut immunity via efferent parasympathetic or sympathetic pathways. Critical unanswered questions include: Do hepatic sensory neurons directly detect inflammatory cytokines, or metabolic stress? How does liver injury—such as cholestasis or steatohepatitis—alter neural signaling to the gut? Can vagal-immune reflexes be pharmacologically or electrically stimulated to treat inflammatory diseases? Addressing these questions will require integrating GCAMP calcium imaging, viral tracing, and Cre-dependent mouse models with immune profiling to map the functional architecture of gut-liver neuroimmune circuits.

Additionally, the molecular mediators orchestrating gut-liver communication remain incompletely characterized. Beyond canonical signals such as BAs, the roles of non-coding RNAs, extracellular vesicles, and intermediate metabolites require systematic investigation. This lack of clarity hampers the identification of novel therapeutic targets. Strategies aimed at modulating the gut microbiota—such as engineered probiotics, or BA supplementation—hold significant promise. However, inter-individual variability poses a major challenge to clinical translation. Factors such as age, metabolic status, and immune responses contribute to this heterogeneity, but population-specific regulatory mechanisms remain insufficiently explored. A standardized framework is needed to reconcile personalized approaches with broader applicability. Finally, the field continues to face a substantial translational gap from bench to bedside, as large-scale, high-quality randomized controlled trials are still lacking. Moving forward, research should prioritize elucidating the liver’s central role in regulating intestinal immunity by constructing multi-layered, cross-tissue regulatory maps. This will not only enhance our understanding of the pathogenesis of chronic inflammation but also lay a theoretical foundation for systemic, gut-liver–targeted therapeutic strategies.
